# Adenine phosphoribosyl transferase deficiency leads to renal allograft dysfunction in kidney transplant recipients: a systematic review

**DOI:** 10.1590/2175-8239-JBN-2021-0283en

**Published:** 2022-05-27

**Authors:** Ishfaq Rashid, Ashish Verma, Pramil Tiwari, Sanjay D’Cruz

**Affiliations:** 1 National Institute of Pharmaceutical Education and Research, Department of Pharmacy Practice, S.A.S. Nagar, 160062, Punjab, India.; 2 Government Medical College and Hospital, Department of General Medicine, 160030, Chandigarh, India.

**Keywords:** Adenina Fosforribosiltransferase, Renal Insufficiency, Adenine Phosphoribosyl Transferase (Aprt) Deficiency, Renal Allograft Dysfunction, 2,8-Dihydroxyadenine, 2,8-Dha Crystal Nephropathy, 2,8-Dihydroxyadeninuria, Renal Replacement Therapy, Adenine Phosphoribosyltransferase, Insuficiência renal, Deficiência de adenine fosforibosil transferase (APRT), disfunção do enxerto renal, 2,8 dihidroxiadenina, Nefropatia por cristal de 2,8 DHA, 2,8 dihidroxiadeninuria, Terapia renal substitutiva

## Abstract

**Background:**

Adenine phosphoribosyl transferase (APRT) deficiency has great implications on graft survival in kidney transplant patients. This systematic review investigated the diagnostic pattern, treatment approach, and kidney transplant outcomes among kidney transplant patients with adenine phosphoribosyl transferase deficiency.

**Material and methods:**

Articles reporting the APRT enzyme deficiency and kidney allograft dysfunction were retrieved from PubMed/Medline, ScienceDirect, Cochrane library and Google scholar databases. Descriptive analysis was used to draw inferences.

**Results:**

The results from 20 selected studies covering 30 patients receiving 39 grafts had an average age of 46.37 years are presented. Graft survival time of more than 6 months was reported in 23 (76.7%) patients, while other 7 (23.3%) patients had graft survival time of less than 6 months. Only 4 (13.3%) patients had APRT deficiency before transplantation. After follow-up, one-third of the patients 10 (33.3%) had stable graft function, 1 patient had allograft loss, 8 (26.6%) patients had delayed graft function while the remaining 11 (36.6%) patients had chronic kidney graft dysfunction.

**Conclusions:**

APRT deficiency is an under-recognized, treatable condition that causes reversible crystalline nephropathy, leading to loss of allograft or allograft dysfunction. The study results showed that inclusion of genetic determination of APRT deficiency in the differential diagnosis of crystalline nephropathy, even in the absence of a history of nephrolithiasis, can improve renal outcomes and may improve allograft survival.

## Resumo

**Antecedentes:**

A deficiência de adenina fosforibosiltransferase (APRT) tem grandes implicações na sobrevida do enxerto em pacientes transplantados renais. Esta revisão sistemática investigou o padrão diagnóstico, a abordagem de tratamento e os desfechos do transplante renal entre pacientes transplantados renais com deficiência de adenina fosforibosiltransferase.

**Material e métodos:**

Os artigos que relatam sobre a enzima APRT e a disfunção do aloenxerto renal foram recuperados do PubMed/Medline, ScienceDirect, Biblioteca Cochrane e bancos de dados do Google Acadêmico. Utilizou-se a análise descritiva para extrair inferências.

**Resultados:**

Foram incluídos participantes que receberam 39 enxertos, a maioria dos quais provenientes de doadores vivos seguidos por doadores falecidos e doadores cadáveres. Foi relatado tempo de sobrevida do enxerto superior a 6 meses em 23 (76,7%) pacientes, enquanto outros 7 (23,3%) pacientes tiveram tempo de sobrevida do enxerto inferior a 6 meses. Apenas 4 (13,3%) pacientes apresentaram deficiência de APRT antes do transplante. Após acompanhamento, um terço dos pacientes, 10 (33,3%) apresentaram função do enxerto estável, 1 paciente teve perda do aloenxerto, 8 (26,6%) pacientes apresentaram função retardada do enxerto, enquanto os 11 (36,6%) pacientes restantes tiveram disfunção crônica do enxerto renal.

**Conclusões:**

A deficiência de APRT é uma causa subestimada e reversível de nefropatia cristalina que leva à disfunção do aloenxerto renal ou à perda total do aloenxerto. Os resultados deste estudo pedem a inclusão desta condição no diagnóstico diferencial de nefropatia cristalina, mesmo na ausência de um histórico de nefrolitíase.

## Introduction

Adenine phosphoribosyl transferase (APRT) deficiency is a rare, autosomal recessive inherited metabolic disorder that generates abundant, poorly soluble 2,8-dihydroxyadenine in urine typically manifesting as recurrent nephrolithiasis, urolithiasis, or crystalline nephropathy, leading to kidney injury and/or kidney failure^1,2^.

This underestimated genetic form of kidney stones and/or kidney failure occurs due to a p.Gln147X mutation in adenine phosphoribosyl transferase gene^3^. This gene (p.Gln147X) is approximately 2.6 kb long, holds five exons and four introns, encodes a protein of 180 amino acid residues^4^, and is positioned on chromosome 16q24^5^.

More than 40 mutations in the coding region of the adenine phosphoribosyl transferase gene were detected in the pathologic allelic variants of 300 affected individuals from more than 25 countries, most of whom (200) were from Japan^3^, but in vitro and in vivo studies revealed that only five allelic variants are currently responsible for the complete inactivation of the APRT protein. APRT gene alterations have been described as frameshift, missense, nonsense mutations and small deletions/insertions ranging in size from 1 to 8 base pairs^3^. This disorder is commonly observed in adults but can be occur in all age groups and stage of disease, with most patients diagnosed either in end-stage kidney failure or after kidney transplantation.

APRT enzyme is linked to Type 1 phosphoribosyl transferase (PRTase) family. It catalyzes the formation of 5'-adenosine monophosphate (5'-AMP) and pyrophosphate (PP) from adenine and 5-phosphoribosyl-1-pyrophosphate.

The conversion of adenine to adenosine is halted in the absence of APRT enzyme activity. Adenine is pushed to metabolize through an alternate pathway where it is transformed into 8-hydroxyadenine and 2,8-dihydroxyadenine (DHA) by xanthine oxidase^6^. DHA is not systemically deposited^7^ and is insoluble at any physiological urine pH. It leads to the formation of 2,8-DHA crystals in the urine, eventually resulting in 2,8-DHA nephrolithiasis and/or crystalline nephropathy. DHA does not normally occur as a metabolic product in humans.

The recurrence of this disease after kidney transplantation and the poor clinical outcomes reported to date for patients is of concern. If recognized early, the treatment with a low-purine diet, high fluid intake, and xanthine analogs can prevent and reverse kidney failure by reducing the formation of 2,8-DHA.

However, it has been reported that the diagnosis of APRT deficiency is extremely variable due to the radiolucent nature of DHA crystals and often misdiagnosed as uric acid stones. Furthermore, the DHA crystals behave the same as oxalate crystals on kidney biopsy, as both are highly birefringent and deposit on tubular cell cytoplasm, tubular lumina, leading to misdiagnosis of oxalosis and chronic kidney disease of unknown etiology^8^.

For patients with urolithiasis or crystal nephropathy, the following diagnostic tests have been used to evaluate the APRT deficiency: (i) APRT gene analysis, (ii) measurement of APRT enzyme in erythrocytes or red cell lysates, (iii) characterization of 2,8-DHA crystals in the urinary sediment using infrared spectroscopy on biopsy, (iv) measurement of adenine levels in a 24-hour urine sample^3^.

APRT deficiency seems to be an important determinant in patients with unexplained nephrolithiasis or crystalline nephropathy. The existing evidence suggests that this metabolic disorder has great implications on graft survival in kidney transplant recipients. In light of this, this review aims to investigate the diagnostic pattern, treatment approach, and outcomes in kidney transplant recipients with APRT deficiency.

## Materials and Methods

### Search Methods and Criteria for Considering Studies

This systematic review followed the Meta-Analysis of Observational Studies in Epidemiology (MOOSE) guidelines^9^ and the protocol has been published in (PROSPERO: CRD42021234784) in the International Prospective Registry of Systematic Reviews. The search strategy was maintained after a thorough consultation with an expert for optimum inclusion sensitivity. To identify the desired articles published since the inception, an extensive literature search was performed in the following databases: PubMed, Cochrane library, Google Scholar, and ScienceDirect. The search strategy was also extended to relevant journals to find out the articles which were not available in databases.

The electronic search on PubMed/Medline database was performed using advanced filters and Medical Science Heading (MeSH) terms: (“Adenine phosphoribosyl transferase (APRT) deficiency”[tiab] OR “recurrent renal stone”[tiab] OR “2,8-dihydroxyadenine”[tiab] OR 2,8-DHA crystal nephropathy OR 2,8-dihydroxyadeninuria OR type I APRT deficiency OR Urolithiasis, 2,8-Dihydroxyadenine) AND (kidney transplantation OR renal transplantation OR Renal transplant patients OR “Renal allograft dysfunction”[tiab] OR “homozygosity”[tiab] OR “crystal-induced kidney damage”[tiab] OR “Renal replacement therapy”[Mesh] OR “Renal transplant*”[tiab] OR “Renal dialysis”[Mesh] OR “Hemodiafiltration”[Mesh] OR “Haemodialysis”[tiab] OR “Peritoneal dialysis”[Mesh] OR “Kidney Grafting”[tiab])). The similarity check for all the selected articles was performed by cross-referencing.

### Inclusion and Exclusion Criteria

**Figure 1:**
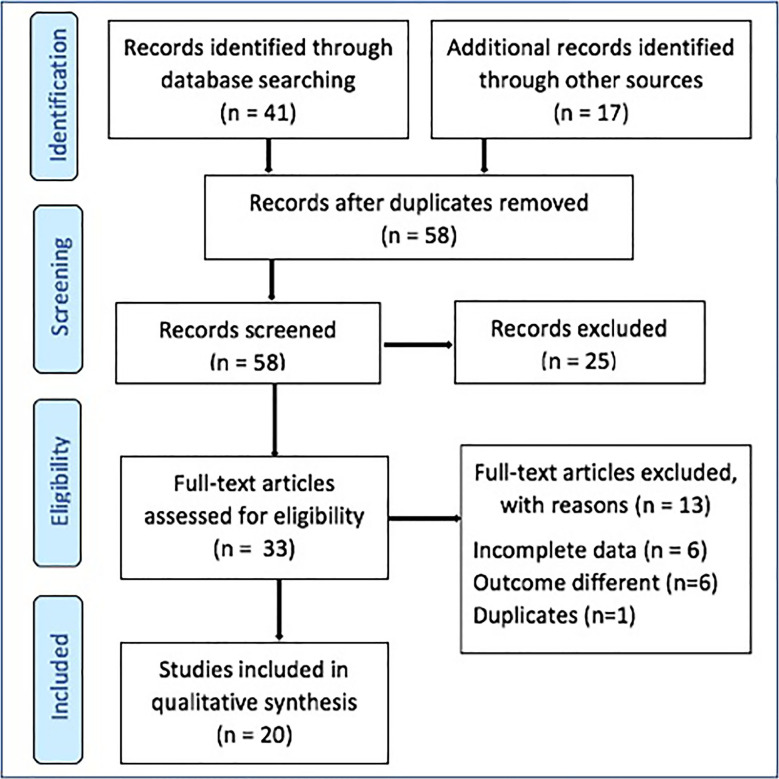
PRISMA (Preferred Reporting Items for Systematic Reviews and Meta-Analysis) flow diagram for studies selection on kidney allograft dysfunction in kidney transplant recipients due to adenine phosphoribosyl transferase deficiency.

Articles on APRT enzyme deficiency in kidney transplant patients from all age groups were selected. The exclusion criteria were unresolved discrepancies in data or data repetition in different studies or unreported APRT deficiency, missing data, duplicates or language barrier [Figure 1].

### Outcomes

Study outcomes included patient demographic characteristics, medical history, diagnosis, nature of 2,8-DHA crystals, treatment approach, and kidney transplant outcomes (number of kidney grafts received, graft survival time, and kidney allograft status).

### Data Extraction and Analysis

All articles retrieved from databases and in the manual search were first independently screened for title and abstract to check for eligibility by two authors [IR and AV]. Then the eligible articles were screened for full text based on study population, sample size, outcome variable, study location etc. by the authors [IR and AV] in accordance with the predefined criteria.

During data extraction phase, two authors [PT, SD] were assigned to handle any sort of discrepancies in the study and data. Articles with incomplete data were excluded. The Microsoft Excel spreadsheet was used to manage the data extracted under the column headings: title of the study, journal name, year of study, study design, 1^st^ author’s name, study location, diagnostic method used, graft survival time, kidney donor, treatment etc. from the eligible articles. Descriptive statistical analysis was employed to draw the inferences.

### Quality Assessment

The Joanna Briggs Institute (JBI) Critical Appraisal tools were used to assess the quality of case reports and case series. The JBI Critical Appraisal tool for case reports has an 8-item checklist that includes patient demographics, history, diagnostic tests or assessment methods, intervention, clinical condition, post-intervention clinical condition, adverse events, and take away message. The JBI Critical Appraisal tool for case reports has 10-item checklist that includes inclusion criteria, valid methods used for identification of the condition, condition measured in a standard way, complete inclusion of participants, patient demographics, consecutive inclusion of participants, clinical information, outcomes, site-specific demographics, and statistical analysis. Scoring was performed using the item checklist and studies who scored more than 50% were included.

## Results

### Study And Patient Characteristics

Forty-one eligible articles were retrieved from MEDLINE/PubMed and seventeen (17) eligible articles from other data sources [Figure 1]. The initial screening based on title and abstract found a total of 58 articles, of which 25 did not meet the inclusion criteria. The final screening based on full text involved the remaining 33 articles, of which 13 articles were excluded due to unwanted outcomes, incomplete data, and duplication. The 20 studies containing 30 samples were considered for final review and qualitative analysis^1,2,7,8,11-26^. The detailed overview of all the 20 eligible studies is provided as Supplementary file.

The majority of the patients was male (66.7%). At the time of diagnosis, the average age of the patients was 46.37 years. Of the 30 patients, 5 were elderly/geriatric (≥ 60 years of age), while 23 were adults (from 18 to 59 years of age), 1 patient was ≤18 years of age, and the data regarding age for 1 patient was not available. The average follow-up was 20.4 months (Table 1).

**Table 1:** Demographic characteristics

Patient characteristics	N (%)	
Average age of the patients at diagnosis	46.37 years	
Mean duration of dialysis before transplantation	2.3 years	
Mean duration of patient follow-up	20.4 months	
Gender	Males	20
	Females	10
Family history of kidney disease	Present	8
	Absent	22
Patient history of kidney disease	Present	28
	Absent	2
Patients on dialysis	Yes	15
	No	15
Study type	Case reports	16
	Case series	4

The patients with APRT deficiency were evaluated for demographic characteristics, medical history, diagnosis, nature of 2,8-DHA crystals, treatment approach, and kidney transplant outcomes (graft survival time and kidney allograft status).

A total of 4 case series and 16 case reports were evaluated. Most of the cases (6 each) were reported from France and Italy, 5 cases were from USA, 3 cases from India, 2 cases from Canada, while 1 case each were from Turkey, Japan, Finland, Germany, UK, and Netherland (Supplementary file).

### Medical History

A total of 8 (26.6%) patients reported family history of kidney disease, 3 of whom had familial APRT deficiency (Table 1). Of the 30 patients, 28 (93.3%) had some level of kidney disease, with 12 (40.0%) patients having a history of chronic kidney failure, 7 (23.3%) having a history of nephrolithiasis, urolithiasis, and kidney staghorn lithiasis, while the remaining 9 (30.0%) had some level of hypertension along with other complications like diabetes, obesity, (3 patients), kidney stones (2 patients), kidney colic (2 patients), pyelonephritis (1 patient), kidney disease not clearly defined (1 patient) (Supplementary file).

### Status of Kidney Disease

Most of the patients (23, 76.6%) had end-stage kidney disease. The average creatinine levels before transplantation were 7.51 mg/dL for 10 samples, while the data for the remaining 20 samples was not available. The post-transplant average creatinine levels were 2.60 mg/dL for 22 samples, while there was no sufficient data for the remaining 8 samples. After the end of follow up, the average creatinine levels were 2.35 mg/dL for 18 samples, while the data for the remaining 12 samples was not available. Fifteen (50.0%) patients were on some form of dialysis before kidney transplant. The dialysis duration was less or equal to 1 year for 6 patients, while it was greater than 1 year for the remaining 9 patients. The data regarding dialysis for the other 15 patients were not available. Insufficient data were available for the genetic characteristics of the majority of the patients, but a few patients (6) were homozygous and heterozygous (3 each). Based on this evidence, the researchers cannot rule out the possibility that APRT deficiency can be genetic.

### Diagnostic Approaches

Diagnosis of APRT deficiency was performed mostly by genetic analysis and graft biopsy. More than half of the patients (16, 53.3%) had genetic analysis of the APRT gene. Graft biopsy analysis was performed alone or in combination with Fourier transform infrared spectroscopy (FTIR), infrared (IR) spectroscopy, high-performance liquid chromatography (HPLC), red blood cell (RBC) assay, chromatography, and X-ray diffraction. FTIR and IR were also performed separately.

Graft biopsy was performed in 9 patients, FTIR in 3 patients, IR spectroscopy in 4 patients. Graft biopsy in combination with FTIR was performed in 7 patients. Kidney biopsy with IR, kidney biopsy with HPLC, kidney biopsy with RBC assay, kidney biopsy with chromatography, and kidney biopsy with X ray diffraction were also performed in 1 patient each.

APRT deficiency was found in 25 patients (83.3%) after kidney transplantation and in 4 patients (13.3%) before transplantation. The data for APRT deficiency was not available for 1 patient (Table 2).

**Table 2:** Diagnostic methods and clinical outcomes of studies on patients with APRT deficiency

	Author	Number of grafts received	Type of donor	Method of analysis		APRT Diagnosis (Pre/Post transplantation)	Graft biopsy Characteristics	Graft survival time (months)	Outcome
				Genetic analysis	Other method				
1	Rajput et al, 2020	1	Kidney transplant from mother	Variant in Exon1 of APRT gene	Graft biopsy	Post	Allograft biopsy was performed, which showed reddish brown crystals in many tubules	> 2 months	Kidney allograft dysfunction
		1	Living donor kidney transplant	Not conducted	Graft biopsy	Post	Allograft biopsy showed reddish brown crystals in the tubules and interstitium. Primary oxalosis was suspected and he was put on treatment with hydration and pyridoxine	> 2 months	Acute graft dysfunction
2	Bagai et al, 2019	1	Underwent live donor kidney allograft transplantation from wife	Anti-sense variation in exon 3 of the APRT gene that resulted in stop codon and premature truncation of the protein at codon 87 so APRT deficiency	Graft biopsy	Post	Graft biopsy was done, which showed normal glomeruli with presence of multiple light brown annular intratubular crystals with surrounding giant cell reaction on light microscopy	> 2 months	Acute graft dysfunction
3	Li et al, 2019	1	Deceased donor kidney transplant	Not conducted	Allograft biopsy	Pre	Biopsy on day 21 showed similar findings (crystal nephropathy, borderline rejection) and he received further pulse methylprednisolone.	> 6 months	DGF
4	George et al, 2017	1	Living-donor kidney transplant from sister	Not conducted	FTIR spectroscopy	Post	NA	Functional state till last follow up	Stable graft
5	Nanumuko et al, 2017	1	Living-related kidney transplantation from mother	A nonsense mutation from TGG to TGA at codon 98	Graft biopsy and IR spectroscopy	Post	Similar small crystal depositions were observed within the tubular lumen in a kidney allograft biopsy specimen obtained on postoperative day 7	> 2 months (110 days)	Stable graft
	Author	Number of grafts received	Type of donor	Method of analysis		APRT Diagnosis (Pre/Post transplantation)	Graft biopsy Characteristics	Graft survival time (months)	Outcome
				Genetic analysis	Other method				
6	Brilland et al, 2015	1	NA	Conducted	NA	Post	No signs of graft rejection on the biopsy; however, ATN with intratubular deposition of typical 2,8-DHA crystals was identified or crystal nephropathy	>18 months	Stable graft
7	Kaartinen et al, 2014	2	Cadaveric kidney transplant (both 1 and 2)	A homozygous mutation in c.188G>A, p.G63D in the APRT gene was found, causing glycine (codon GGC) transformation into aspartate.	Graft biopsy	Post	Allograft biopsy was taken three times: 10,19, and 38 days post-transplantation and biopsy revealed normal glomerular pattern and no signs of rejection but extensive acute tubular injury and intratubular obstruction by needle shaped crystals of unknown type	> 11 months	kKdney allograft dysfunction
8	Quaglia et al, 2014	1	NA	Analysis of the APRT gene showed heterozygous mutation (one base insertion with a frameshift)	Infrared spectrometry	NA	Histological examination showed diffuse intratubular obstruction by crystals identified as 2,8-DHA	NA	DGF
		1	Deceased donor kidney transplant	Not conducted	Allograft biopsy	Pre	1st biopsy - showed mild mesangial proliferation with granular diffuse IgA and C3 deposits and some scanty intratubular crystals assumed as, 2nd biopsy - persistence of mild IgA nephropathy, interstitial inflammation, and diffuse deposits of intratubular brown crystal, 3rd biopsy - showed persistence of intratubular brown crystals with interstitial inflammatory infiltration and fibrosis uric acid	36 months (3 years) The patient died the following year of breast carcinoma.	DGF
	Author	Number of grafts received	Type of donor	Method of analysis		APRT Diagnosis (Pre/Post transplantation)	Graft biopsy Characteristics	Graft survival time (months)	Outcome
				Genetic analysis	Other method				
9	Zaiden et al, 2014	1	All patient except one, received a deceased donor kidney	1st allele exon-4, 2nd allele exon - 4	Graft biopsy and FTIR	Post	Positive crystal on previous graft biopsy and oxalate crystalline nephropathy on current biopsy	>132 months	Chronic graft dysfunction
		1		1st allele exon-3, 2nd allele exon - 3	Graft biopsy and FTIR	Post	Positive crystal on previous graft biopsy and oxalate crystalline nephropathy on current biopsy	>6 months	Chronic graft dysfunction
		2		Not conducted	Graft biopsy and FTIR	Post	Positive crystal on previous graft biopsy and undetermined crystalline nephropathy on current biopsy	>40 months	Stable graft
		2		1st allele exon-1, 2nd allele exon - 4	FTIR	Post	Oxalate crystalline nephropathy on current biopsy	> 24 months	Chronic graft dysfunction
		1		Not conducted	Graft biopsy and FTIR	Post	Positive crystal on previous graft biopsy and urate crystalline nephropathy on current biopsy	>30 months	Chronic graft dysfunction
		1		1st allele exon 5-, 2nd allele exon - undetermined	FTIR	Post	2,8-DHA crystalline nephropathy on current biopsy	> 24 months	Stable graft
		1		1st allele exon-4, 2nd allele exon - undetermined	Graft biopsy and FTIR	Post	Undetermined crystalline nephropathy on current biopsy	8 months	Graft loss
10	Sharma et al, 2012	1	Deceased donor kidney transplant	Not conducted	Allograft biopsy	Post	Kidney allograft biopsy revealed a severe crystalline nephropathy with abundant tubular and interstitial brown, birefringent crystals	1 month (>25 days)	DGF
11	Bertram et al, 2010	3	Third transplant (Deceased donor)	Not conducted	Infrared spectrometry	Post	Allograft biopsy on day 7 revealed acute humoral rejection and intratubular DHA crystals, biopsy on day 23, no signs of acute rejection were found, and the number of DHA crystals had receded	9 months (patient died with Pulmonal-aspergillosis with a functioning graft)	Stable graft
	Author	Number of grafts received	Type of donor	Method of analysis		APRT Diagnosis (Pre/Post transplantation)	Graft biopsy Characteristics	Graft survival time (months)	Outcome
				Genetic analysis	Other method				
12	Micheli et al, 2010	1	Deceased donor kidney transplant	A homozygous C>G substitution at -3 in the splicing site of exon 2 (IVS2 -3 c>g)	X-ray diffraction and APRT activity was measured in lysates and in intact erythrocytes	Post	Post-transplant performed and disclosed interstitial crystal deposition with diffuse intratubular crystal cast formations	60 months (> 5 years)	Stable graft
13	Nasr et al, 2010	2	Deceased donor kidney transplant (1 and 2 both)	Not conducted	Allograft biopsy and X-ray microanalysis of the tubular crystals	Post	Recurrent disease was documented in allograft biopsies performed 3 days, 4 months, 5 months, 8 months and 1 year post-transplant. None of the biopsies showed acute rejection or calcineurin inhibitor toxicity	12 months (> 1 year)	KGD
		1	Living related kidney transplant	Not conducted	Allograft biopsy	Pre	Post transplantation allograft biopsy after 2 month revealed acute tubular necrosis but no crystals were identified	4 months	Stable graft
		1	Deceased donor kidney transplant	Not conducted	Allograft biopsy	Pre	A repeat biopsy at 3 months post-transplant showed fewer crystals with minimal tubular atrophy, interstitial fibrosis and inflammation	> 18 months	DGF
14	Stratta et al, 2010	1	Deceased donor kidney transplant	This analysis showed only 1 heterozygous sequence variant, a duplication of T at position 1832 in the genomic DNA, resulting in deletion of exon 4 in messenger RNA, premature termination at amino acid 110, and a truncated protein of 109 amino acids instead of 180	Radiolabeled 14C-adenine in a chromatographic assay for APRT activity and allograft biopsy	Post	1st biopsy post transplantation - normal glomerular, interstitial, and vascular morphologic patterns, but intratubular obstruction by crystals of unknown type, second biopsy performed 2 weeks later, and again showing many brown and irregular needle-shaped intratubular crystals	12 months (> 1 year)	DGF
	Author	Number of grafts received	Type of donor	Method of analysis		APRT Diagnosis (Pre/Post transplantation)	Graft biopsy Characteristics	Graft survival time (months)	Outcome
				Genetic analysis	Other method				
15	Cassidy et al, 2004	1	Cadaveric kidney transplant	Not conducted	Infrared spectroscopy	Post	Chronic interstitial nephritis with non-specific crystal deposition.	> 8 months	DGF
16	Eller et al, 2004	4	Cadaveric kidney transplant	hom. c.400 þ2dup	Graft biopsy and FTIR	Post	NA	> 7 months	DGF
17	Benedetto et al, 2001	1	Living-related kidney transplant	Not conducted	Allograft biopsy and erythrocyte assay	Post	Allograft biopsy showed moderate-to-severe chronic interstitial nephritis with marked intratubular and parenchymal deposition of highly birefringent needle-shaped crystals arranged in an annular pattern. These crystals were suggestive of 2,8-DHA interstitial nephritis secondary to APRT deficiency.	>19 months	Kidney allograft dysfunction
18	Brown et al, 1998	1	Cadaveric kidney transplant	Not conducted	Infrared spectrometry	Post	A kidney biopsy 14 days post-transplant showed acute tubular necrosis	> 3 months (132 days)	Stable graft
19	De jong et al, 1996	1	NA	Not conducted	Kidney biopsy and HPLC in urine and serum for detection of 2,8-dihydroxyadenine	Post	Biopsy of the kidney graft was obtained on day 10 postoperatively because of oliguria. Little sign of rejection or cyclosporine nephrotoxicity was noted	6 months	Stable graft
20	Gagne et al, 1994	1	Cadaveric kidney transplant	Not conducted	Kidney biopsy and Fourier transform infrared microscopy	Post	A biopsy of the allograft was performed that showed severe chronic interstitial nephritis associated with marked intratubular and parenchymatous deposition of highly birefringent needle-shaped crystals very similar to those observed on the native kidney	108 months (> 9 years)	KGD

### Nature of 2,8-Dha Crystals

Allograft biopsy revealed different morphological characteristics of 2,8-DHA crystals: a normal glomerulus with multiple light brown annular intratubular crystals deposited within the tubular lumen, reddish brown crystals in several tubules and interstitium, acute tubular necrosis with intratubular deposition and/or obstruction of needle-shaped and typical 2,8-DHA crystals. However, in some patients, graft biopsy showed mild mesangial proliferation with granular diffuse immunoglobulin A (IgA) and complement 3 (C3) deposits and some scanty intratubular crystals. Also, persistent mild IgA nephropathy, intratubular brown crystals with interstitial inflammatory infiltration, and fibrosis uric acid were noticed. Oxalate crystalline nephropathy with abundant tubular and interstitial brown, birefringent crystals, minimal tubular atrophy, and interstitial crystal deposition with diffuse intratubular crystal cast formations were also seen on graft biopsy analysis.

Allograft biopsy also showed moderate-to-severe chronic interstitial nephritis with noticeable intratubular and parenchymal deposition of highly birefringent needle-shaped crystals arranged in an annular pattern (Table 2).

### Clinical Outcomes: Grafts Received, Graft Survival Time and Allograft Status

The number of kidney grafts (39) received exceeds the number of patients (30) enrolled for analysis. The majority of the patients (24, 80.0%) received 1 graft, 4 patients received 2 grafts each, 1 patient received 3 grafts, and 1 received 4 grafts. Kidney donor details were available for 36 grafts. Ten grafts were from living donors, 17 were from deceased donors, and 9 were from cadaveric donors.

Graft survival time was reported in months. The majority of the patients (23, 76.7%) had more than 6 months of graft survival while 7 (23.3%) had less than 6 months of graft survival. The shortest graft survival time was 1 month and the longest was 132 months. The average survival time was 19.5±30.5 months (mean with standard deviation).

After follow-up, one-third of the patients (10, 33.3%) had stable graft status, 1 patient had graft loss, 8 patients (26.6%) had delayed graft function, and 11 patients (36.6%) had kidney graft dysfunction (Table 2).

### Treatment Approach

The patients were treated with 3 different therapeutic options: xanthine oxidase enzyme inhibitors (allopurinol 150, 200, 300, 400, and 500 mg and febuxostat 20, 40, and 80 mg), immunosuppressants (Induction therapy - basiliximab, Maintenance therapy - prednisolone + mycophenolate sodium + cyclosprine/methylprednisolone/tacrolimus), and hydration and low-purine diet. A large proportion of patients (28, 93.3%) were treated with allopurinol after kidney transplant, and data for the remaining 2 (6.4%) patients were not available. Around one-third of the patients (2, 66.3%) were treated with immunosuppressants, of whom 10 (33.3%) were on both induction and maintenance therapy and the other 10 (33.3%) only on maintenance therapy. The data regarding treatment for the other 10 (33.4%) patients were not available. Also, 9 patients (30.0%) were on hydration and low-purine diet, and the data regarding 15 patients (50.0%) were not available, while the other 6 patients (20.30%) had not received any hydration and low-purine diet (Supplementary file).

### Quality Assessment

Ten case-reports had a score of 7 on the JBI Critical Appraisal tool (of a total of 8), two studies had a score of 8, and 4 studies had a score of 6. For case series, three (3) studies had a score of 7 points while one (1) study had a score of 9 points (of a total of 10) (Supplementary file).

## Discussion

Twenty studies with a total of 30 patients with recurrent 2,8-DHA crystalline nephropathy were included in the review. In most of the cases 25(83.3%), APRT deficiency was diagnosed after kidney transplantation. This systematic review aimed to provide a valuable overview of this rare inherited metabolic disorder in the context of kidney transplantation, emphasizing the need for a comprehensive understanding on APRT deficiency among all the healthcare stakeholders.

The diagnosis of APRT deficiency usually remains unnoticed for years, which poses a great challenge in its management, especially when there is no clinical suspicion. This is also supported by the result of this study, as around 83.3% of patients were diagnosed with APRT deficiency after kidney transplantation.

The genetic testing confirms APRT deficiency by the presence of significant functional mutations in both alleles. Since sequencing of exons and flanking intronic sequences of the APRT gene permits identifying about 90% of mutations^27^, the mutation analysis of APRT gene can be used as first-line test for the diagnosis of this rare metabolic disorder when other techniques are not considered or unavailable. The findings of this study support this statement, as genetic analysis of APRT deficiency was performed in more than half of the patients.

One of the important determinants for the diagnosis of crystalline nephropathy comes by defining the nature of crystals detected in kidney biopsy. This is in consonance with APRT deficiency, as the presence of 2,8-DHA crystals is pathognomonic of the disease. In this study, different approaches were adopted to describe the nature of 2,8-DHA.

Due to their rarity, DHA crystals are often confused with oxalate, urate, and cystine crystals, and evidence suggests that this disease is gravely underdiagnosed^28^. In this review, different morphological characteristics of 2,8-DHA crystals were described. The presence of birefringence, and staining characteristics like multiple brownish-green or brown stains with H&E and PAS, light blue stains with TRI, and black stains with JMS. The crystals were found to be needle-, rod- or rhomboid-shaped, present alone, as annular formations of striated crystals, or as fan-like or irregular clusters. These crystals show a giant cell reaction and are distributed mainly in tubular lumina and cytoplasm and sometimes in the interstitium (more abundant in the cortex). In some patients, 2,8-DHA crystals had moderate-to-severe chronic interstitial nephritis with marked intratubular and parenchymal deposition of highly birefringent needle-shaped crystals, minimal tubular atrophy, acute tubular necrosis, and interstitial fibrosis. This study also revealed a unique characteristic of 2,8-DHA crystals in few allograft biopsies of patients with recurrent DHA crystalline nephropathy^16^.

The results also highlighted that Fourier transform infrared microscopy (FTIR) combined with polarized light microscopy can better characterize crystals in kidney biopsies than examination with and polarized light microscopy alone^29^.

One-third of the grafts were from living donors while the rest were from deceased donors. The kidney grafts received by the patients were quite higher compared with the number of participants, which suggests that graft rejection is quite common in these patients. Graft survival time ranged from 1 month to 132 months and the majority (76.7%) of patients had more than 6 months of graft survival. These findings are consistent with those of Runolfsdottir et al. who concluded that timely diagnosis and treatment with xanthine oxidoreductase inhibitor therapy before transplant can improve allograft outcomes like graft survival time, while delay in such treatment has proven to be a frequent cause of premature loss of allograft^30^].

After the follow-up, one-third of the patients had stable graft status, while more than one-third of the patients had kidney graft dysfunction, which shows that allograft dysfunction is quite common among these patients.

The management of this metabolic disorder involves the reduction of systemic dihydroxyadenine production by decreasing purine intake (although most production occurs via salvage pathway) and inhibiting xanthine oxidase/dehydrogenase with allopurinol or febuxostat (a non-purine selective inhibitor), which is main therapy of APRT deficiency. In this study, 93.3% of patients were treated with allopurinol after the kidney transplant. This is supported by the findings by Bollee et al. and Harambat et al. who reported that most of the adult patients treated with 200-600mg per day and children treated with 5-10 mg/kg per day dramatically depletes the generation of crystals^31,32^. Febuxostat is preferred in patients allergic to allopurinol^33^.

The timely management of this metabolic disorder with the xanthine dehydrogenase inhibitor allopurinol reduces the formation of crystals in the urine thus ameliorating the rate of graft loss^34^. Patient adherence to treatment plays an important role in APRT deficiency. As a sustained fall of crystals in the urine is expected during treatment, repeated quantitative analysis of crystals in the urine can be a useful guide for the perfect management of this disease.

Treatment with high doses of allopurinol (up to 600 mg)^30,16,2^ and febuxostat^35^ (80 mg) is mandatory to achieve the effective inhibition of dihydroxyadenine crystals in the urine. Dose titration (upward) is requisite if satisfactory response is not obtained with the starting dose and also if patients with DHA nephropathy relapse^2^.

Immunosuppressants play a pivotal role in graft rejection. In this study, around one-third of patients were treated with immunosuppressants combined with the mainstay therapy with allopurinol. Pregnant patients require special attention on usage of immunosuppressant drugs (post transplantation) as these drugs are known to have adverse effects on the developing fetus.

Adequate fluid intake and low-purine diet are also advised. Nine patients in this study were treated with hydration and low-purine diet. A fluid intake of at least 2.5-3.0 litres daily for adults should be encouraged, but special caution is required for dialysis patients as a high fluid intake is contraindicated. A low-purine diet should decrease the 2,8-DHA excretion. Urine alkalinization is also contraindicated as 2,8-DHA crystals are very insoluble at high pH.

## Conclusions

In conclusion, this rare autosomal recessive metabolic disorder is an underrated condition and a reversible cause of crystalline nephropathy, which leads to graft rejection. This review provides all healthcare stakeholders with a detailed overview of the demographics, diagnosis and diagnostic tests, morphology of 2,8-DHA crystals, treatment approaches, outcomes like frequency of grafts received, graft survival time, and kidney allograft status in patients with APRT deficiency receiving kidney transplant.

Due to its rare nature and lack of long-term studies, this detailed description of APRT deficiency in kidney transplant recipients may enable all healthcare stakeholders to apply the necessary diagnostic and therapeutic approaches like XOR inhibitors before or at the time of kidney transplantation to achieve good clinical outcomes. It may also help them better understand the risk of relapse of crystalline nephropathy in kidney transplant recipients with APRT deficiency.

As it has been reported, delayed diagnosis of APRT deficiency is a leading cause of premature graft loss in these patients. Therefore, APRT deficiency should be included in the differential diagnosis of crystalline nephropathy even with no history of nephrolithiasis, as a timely diagnosis has therapeutic implications.

### Limitations of the Study

The data regarding long term outcomes associated with APRT deficiency after kidney transplantation are scarce and the availability of diagnostic tests also varies from region to region. As there are only case reports and case series available on this topic, lack of sufficient data has allowed us to conduct only a systematic review.

## Supplementary Material

The following online material is available for this article:

Supplement A - Study and patient characteristics.

Supplement B - Quality assessment of studies and treatment approach of study participants.

## References

[1] Kaartinen K, Hemmilä U, Salmela K, Räisänen-Sokolowski A, Kouri T, Mäkelä S. (2014). Adenine phosphoribosyltransferase deficiency as a rare cause of renal allograft dysfunction. J Am Soc Nephrol.

[2] Nasr SH, Sethi S, Cornell LD, Milliner DS, Boelkins M, Broviac J (2010). Crystalline nephropathy due to 2,8-dihydroxyadeninuria: an under-recognized cause of irreversible renal failure. Nephrol Dial Transplant.

[3] Valaperta R, Rizzo V, Lombardi F, Verdelli C, Piccoli M, Ghiroldi A (2014). Adenine phosphoribosyltransferase (APRT) deficiency: identification of a novel nonsense mutation. BMC Nephrol.

[4] Broderick TP, Schaff DA, Bertino AM, Dush MK, Tischfield JA, Stambrook PJ. (1987). Comparative anatomy of the human APRT gene and enzyme: nucleotide sequence divergence and conservation of a nonrandom CpG dinucleotide arrangement. Proc Natl Acad Sci U S A.

[5] Tischfield JA, Ruddle FH. (1974). Assignment of the gene for adenine phosphoribosyltransferase to human chromosome 16 by mouse-human somatic cell hybridization. Proc Natl Acad Sci U S A.

[6] WYNGAARDEN JB, DUNN JT (1957). 8-Hydroxyadenine as the intermediate in the oxidation of adenine to 2, 8-dihydroxyadenine by xanthine oxidase. Arch Biochem Biophys.

[7] Li J, Shingde M, Nankivell BJ, Tchan MC, Bose B, Chapman JR (2019). Adenine Phosphoribosyltransferase Deficiency: A Potentially Reversible Cause of CKD. Kidney Int Rep.

[8] Rajput P, Virani ZA, Shah BV. (2020). Crystalline Nephropathy due to APRT Deficiency: A Preventable Cause of Renal and Renal Allograft Failure. Indian J Nephrol.

[9] Stroup DF, Berlin JA, Morton SC, Olkin A, Williamson GD, Rennie D (2000). Meta analysis of Observational Studies in Epidemiology: A Proposal for Reporting. JAMA.

[10] Page MJ, McKenzie JE, Bossuyt PM, Boutron I, Hoffmann TC, Mulrow CD (2021). The PRISMA 2020 statement: an updated guideline for reporting systematic reviews. BMJ.

[11] Bagai S, Khullar D, Bansal B. (2019). Rare crystalline nephropathy leading to acute graft dysfunction: a case report. BMC Nephrol.

[12] George SA, Al-Rushaidan S, Francis I, Soonowala D, Nampoory MRN. (2017). 2,8-Dihydroxyadenine Nephropathy Identified as Cause of End-Stage Renal Disease After Renal Transplant. Exp Clin Transplant.

[13] Nanmoku K, Kurosawa A, Shinzato T, Shimizu T, Kimura T, Yagisawa T. (2017). Febuxostat for the Prevention of Recurrent 2,8-dihydroxyadenine Nephropathy due to Adenine Phosphoribosyltransferase Deficiency Following Kidney Transplantation. Intern Med.

[14] Brilland B, Augusto JF, Croue A, Subra JF, Sayegh J. (2015). A rare case of primary non-function of renal allograft due to adenine phosphoribosyltransferase deficiency. Int Urol Nephrol.

[15] Quaglia M, Musetti C, Ghiggeri GM, Fogazzi GB, Settanni F, Boldorini RL (2014). Unexpectedly high prevalence of rare genetic disorders in kidney transplant recipients with an unknown causal nephropathy. Clin Transplant.

[16] Zaidan M, Palsson R, Merieau E, Cornec-Le Gall E, Garstka A, Maggiore U (2014). Recurrent 2,8-dihydroxyadenine nephropathy: a rare but preventable cause of renal allograft failure. Am J Transplant.

[17] Sharma SG, Moritz MJ, Markowitz GS. (2012). 2,8-dihydroxyadeninuria disease. Kidney Int.

[18] Bertram A, Broecker V, Lehner F, Schwarz A. (2010). Kidney transplantation in a patient with severe adenine phosphoribosyl transferase deficiency: obstacles and pitfalls. Transplant International.

[19] Micheli V, Massarino F, Jacomelli G, Bertelli M, Corradi MR, Guerrini A (2010). Adenine phosphoribosyltransferase (APRT) deficiency: a new genetic mutation with early recurrent renal stone disease in kidney transplantation. NDT Plus.

[20] Stratta P, Fogazzi GB, Canavese C, Airoldi A, Fenoglio R, Bozzola C (2010). Decreased kidney function and crystal deposition in the tubules after kidney transplant. Am J Kidney Dis.

[21] Cassidy MJ, McCulloch T, Fairbanks LD, Simmonds HA. (2004). Diagnosis of adenine phosphoribosyltransferase deficiency as the underlying cause of renal failure in a renal transplant recipient. Nephrol Dial Transplant.

[22] Eller P, Rosenkranz AR, Mark W, Theurl I, Laufer J, Lhotta K. (2004). Four consecutive renal transplantations in a patient with adenine phosphoribosyltransferase deficiency. Clin Nephrol.

[23] Benedetto B, Madden R, Kurbanov A, Braden G, Freeman J, Lipkowitz GS. (2001). Adenine phosphoribosyltransferase deficiency and renal allograft dysfunction. Am J Kidney Dis.

[24] Brown HA. (1998). Recurrence of 2,8-dihydroxyadenine tubulointerstitial lesions in a kidney transplant recipient with a primary presentation of chronic renal failure. Nephrol Dial Transplant.

[25] De Jong DJ, Assmann KJ, De Abreu RA, Monnens LA, van Liebergen FJ, Dijkman HB (1996). 2,8-Dihydroxyadenine stone formation in a renal transplant recipient due to adenine phosphoribosyltransferase deficiency. J Urol.

[26] Gagné ER, Deland E, Daudon M, Noël LH, Nawar T. (1994). Chronic renal failure secondary to 2,8-dihydroxyadenine deposition: the first report of recurrence in a kidney transplant. Am J Kidney Dis.

[27] Ceballos-Picot I, Daudon M, Harambat J, Bensman A, Knebelmann B, Bollee G. (2014). 2,8-dihydroxyadenine urolithiasis: a not so rare inborn error of purine metabolism. Nucleosides, Nucleotides Nucleic Acids.

[28] Ceballos-Picot I, Perignon JL, Hamet M, Daudon M, Kamoun P. (1994). 2,8-Dihydroxyadenine urolithiasis, an underdiagnosed disease. Lancet.

[29] Dessombz A, Bazin D, Dumas P, Sandt C, Sule-Suso J, Daudon M. (2011). Shedding light on the chemical diversity of ectopic calcifications in kidney tissues: diagnostic and research aspects. PLoS One.

[30] Runolfsdottir HL, Palsson R, Agustsdottir IMS, Indridason OS, Li J, Dao M (2020). Kidney Transplant Outcomes in Patients With Adenine Phosphoribosyltransferase Deficiency. Transplantation.

[31] Bollee G, Dollinger C, Boutaud L, Guillemot D, Bensman A, Harambat J (2010). Phenotype and genotype characterization of adenine phosphoribosyltransferase deficiency. J Am Soc Nephrol.

[32] Harambat J, Bollee G, Daudon M, Ceballos-Picot I, Bensman A. (2012). Adenine Phosphoribosyltransferase deficiency in children. Pediatr Nephrol.

[33] Arnadottir M. (2014). Febuxostat in adenosine phosphoribosyltransferase deficiency. Am J Kidney Dis.

[34] Bollée G, Harambat J, Bensman A, Knebelmann B, Daudon M, Cebbalos-Picot I. (2012). Adenine phosphoribosyltransferase deficiency. Clin J Am Soc Nephrol.

[35] Nanmoku K, Kurosawa A, Shinzato T, Shimizu T, Kimura T, Yagisawa T. (2017). Febuxostat for the Prevention of Recurrent 2,8-dihydroxyadenine Nephropathy due to Adenine Phosphoribosyltransferase Deficiency Following Kidney Transplantation. Intern Med.

