# Supplementary material for: Adenine phosphoribosyl transferase deficiency leads to renal allograft dysfunction in kidney transplant recipients: a systematic review
Source: J Bras Nefrol. 2022 May 27;44(3):403–16. doi: 10.1590/2175-8239-JBN-2021-0283en (PMC9518620; doi:10.1590/2175-8239-JBN-2021-0283en)
Supplement: Supplementary file 1 [file 2175-8239-jbn-2021-0283-suppl1.pdf]

Braz. J. Nephrol. (J. Bras. Nefrol.)

https://doi.org/10.1590/2175-8239-JBN-2021-0283

Supplementary Material to "Adenine phosphoribosyl transferase deficiency lead renal allograft dysfunction among kidney transplant recipients: a systematic review"

Supplement A - Study and patient characteristics.

	Author	Type of study	No of Cases	Sex	Age at diagnosis (Years)	Age at Transplantation (Years)	Patient follow up (months)	Family history of kidney disease	Patient history of kidney disease	Status of kidney disease	Duration of dialysis before transplant (Years)
**1**	Rajput et al, 2020, India	Case series	2	M	43	43	2	NA	kidney stones in the past	ESRD	1
				M	31	31	2	NA	History of CKD due to bilateral kidney stones	Haemodialysis	1
**2**	Bagai et al, 2019, India	Case report	1	M	44	44	NA	History of nephrolithiasis	CKD	ESRD	1
**3**	Li et al, 2019, Australia	Case report	1	M	47	52	6	No	Obesity, hypertension, atrial fibrillation	Dialysis	4
**4**	George et al, 2017, Turkey	Case report	1	F	41	41	NA	Yes, her mother and 1 sister had frequent urinary stones	Significant for recurrent nephrolithiasis and urinary tract infections since childhood	ESRD	NA
**5**	Nanumuko et al, 2017, Japan	Case report	1	M	28	28	4	No	Several episodes of urinary stone excretion in childhood	Haemodialysis	5
**6**	Brilland et al, 2015, France	Case report	1	F	25	30	18	NA	Recurrent urinary infections, one episode of renal colic in childhood	ESRD	3
**7**	Kaartinen et al, 2014, Finland	Case report	1	M	63	(1st) 60, (2nd) 63	11	Grandfather had urinary stones	Yes	ESRD	2
**8**	Quaglia et al, 2014, Italy	Case series	2	F	67	67	NA	NA	NA	ESRD	NA
				F	NA	48	36	No	No	ESRD	1
**9**	Zaiden et al, 2014, France (5), Italy (2)	Case series	7	F	28	28	132	NA	CKD due to chronic tubulointerstitial nephropathy	CKD	NA

	Author	Type of study	No of Cases	Sex	Age at diagnosis (Years)	Age at Transplantation (Years)	Patient follow up (months)	Family history of kidney disease	Patient history of kidney disease	Status of kidney disease	Duration of dialysis before transplant (Years)
				M	41	38	6	NA	CKD due to unknown cause	CKD	NA
				M	48	46	40	NA	CKD due to unknown cause	CKD	NA
				M	48	48	24	NA	CKD due to Oxalate nephropathy	Haemodialysis	NA
				F	49	46	30	NA	CKD due to unknown cause	CKD	NA
				F	58	58	24	NA	CKD due to chronic tubulointerstitial nephropathy	CKD	NA
				M	64	64	8	NA	CKD due to unknown cause	Haemodialysis	NA
**10**	Sharma et al, 2012, USA	Case report	1	M	80	80	1	NA	Seizure disorder, and multiple episodes of nephrolithiasis prior 43 years of age	ESRD	3
**11**	Bertram et al, 2010, Germany	Case report	1	M	55	39 (1st), 55 (2nd), 56 (3rd)	9	Yes	Recurrent nephrolithiasis	ESRD	NA
**12**	Micheli et al, 2010, Italy	Case report	1	M	48	43	2	No	Recurrent renal staghorn lithiasis on both sides since the age of 17	Haemodialysis	1
**13**	Nasr et al, 2010, USA	Case series	3	F	42	39	12	Younger sibling is a case of renal failure and deficiency of APRT	History of type 2 diabetes and depression, severe renal failure and anemia	Haemodialysis	3
				M	41	41	4	Elder sibling is a case of renal failure and deficiency of APRT	Hypertension and diet-controlled type 2 diabetes mellitus, acute and chronic tubulointerstitial nephritis and arteriolar hyalinosis.	Haemodialysis	NA
				M	55	54	18	Negative	Type 2 diabetes for 8 years, hypertension and obesity. At the age of 18, he reported kidney stones	Dialysis	1
**14**	Stratta et al, 2010, Italy	Case report	1	F	67	67	12	Family history was negative except for hypertension	Repeated renal colic with recurrent episodes of spontaneous elimination of small kidney stones since childhood	ESRD	2

	Author	Type of study	No of Cases	Sex	Age at diagnosis (Years)	Age at Transplantation (Years)	Patient follow up (months)	Family history of kidney disease	Patient history of kidney disease	Status of kidney disease	Duration of dialysis before transplant (Years)
**15**	Cassidy et al, 2004, London	Case report	1	M	23	23	8	Mother and father are carriers of APRT deficiency	Chronic renal failure of unknown cause	CKD	NA
**16**	Eller et al, 2004, NA	Case report	1	M	11	NA	7	NA	History of nephrolithiasis, CKD	Haemodialysis	NA
**17**	Benedetto et al, 2001, USA	Case report	1	M	44	43	19	NA	Two episodes of urolithiasis	ESRD	NA
**18**	Brown et al, 1998, Canada	Case report	1	F	47	47	4	NA	Episode of pyelonephritis	ESRD	2
**19**	De jong et al, 1996, Netherland	Case report	1	M	56	56	6	NA	History of recurrent radiolucent and radiopaque urolithiasis of supposed uric acid composition	Haemodialysis	NA
**20**	Gagne et al, 1994, Canada	Case report	1	M	51	42	108	NA	History of urolithiasis at the age of 8 years.	ESRD	5
	M = Male, F = Female. ESRD = End Stage Renal Disease. NA = Not available. CKD = chronic kidney disease										
