# Supplementary material for: Adenine phosphoribosyl transferase deficiency leads to renal allograft dysfunction in kidney transplant recipients: a systematic review
Source: J Bras Nefrol. 2022 May 27;44(3):403–16. doi: 10.1590/2175-8239-JBN-2021-0283en (PMC9518620; doi:10.1590/2175-8239-JBN-2021-0283en)
Supplement: Supplementary file 2 [file 2175-8239-jbn-2021-0283-suppl2.pdf]

Braz. J. Nephrol. (J. Bras. Nefrol.)

https://doi.org/10.1590/2175-8239-JBN-2021-0283

Supplementary Material to "Adenine phosphoribosyl transferase deficiency lead renal allograft dysfunction among kidney transplant recipients: a systematic review"

Supplement B - Quality assessment of studies and treatment approach of study participants

	Author	Kidney function or Serum Creatinine (mg/dl)			Quality assessment score	Treatment with XOR inhibitors	Immunosuppressant treatment	Hydration and purine-based diet	Comments
		Pre (Tx) Transplant	Post (Tx) Transplant	End of follow up					
1	Rajput et al, 2020	NA	1.3	NA	7	Treated with allopurinol post Tx	MT- Prednisolone + Mycophenolate sodium + Tacrolimus	NA	NA
		3	1.0(Post Tx), 1.8 (after 2-month Tx), 3.8 (after several months before induction of allopurinol)	NA		Allopurinol (300 mg/day) post Tx	NA	NA	NA
2	Bagai et al, 2019	NA	1.0 - 1.1 mg/dL	2.0 (2 month)	7	Allopurinol 200mg daily post Tx	IT - Basiliximab, MT - Tacrolimus + Mycophenolate mofetil + Steroids	Yes	He attained a baseline serum creatinine within 1 month of therapy. His Uric acid level before initiation of therapy was 7 mg/dl and maintained between 5 and 6 mg/dl on therapy.
3	Li et al, 2019	NA	2.05 mg/dL (180 micromol/l) (3 weeks)	1.85 mg/dL (164 micromol/l) (6 months post Tx)	8	Pre Tx - 150 mg (for 4.5 year), PTx- allopurinol increased up to 400 mg daily post transplantation for 3 weeks and after this 20 mg of Febuxostat was introduced, + allopurinol 600 mg daily.	Maintenance immunosuppression - Tacrolimus, Mycophenolate, prednisolone	No	Four-months after transplantation, he developed invasive cytomegalovirus (CMV) disease
4	George et al, 2017	NA	NA	NA	7	allopurinol (300 mg/day) post Tx	IT- basiliximab, MT- Prednisolone + Mycophenolate sodium + Cyclosporine	NA	At the last follow-up, she had stable renal function and no evidence of crystalluria.
5	Nanumuko et al, 2017	NA	1.64 mg/dL	1.65 mg/dL	7	After 7 day of post-transplant - Febuxostat 20 mg/day. After 14 days of post-transplant - 40mg/day, At 110 day of post-transplant - 80 mg/day	Induction therapy - Basiliximab, Maintenance therapy - Tacrolimus (ER) + Mycophenolate mofetil + Methylprednisolone	Yes	Second renal allograft biopsy & urinalysis on postoperative day demonstrated the absence of 2,8-DHA crystals. Renal allograft function remained stable with Febuxostat 80 mg/day.
6	Brilland et al, 2015	1.1 mg/dL	NA	NA	8	Post Tx day 24, allopurinol (200 mg/day)	(Post Tx) IT- basiliximab, MT-	NA	This case is the first reporting long-term stabilization of

	Author	Kidney function or Serum Creatinine (mg/dl)			Quality assessment score	Treatment with XOR inhibitors	Immunosuppressant treatment	Hydration and purine-based diet	Comments
		Pre (Tx) Transplant	Post (Tx) Transplant	End of follow up					
							Prednisolone + Mycophenolate mofetil + Cyclosporine		kidney function in this setting and there are 3 mismatches (a 3-HLA A-B-DR-mismatched donor)
**7**	Kaartinen et al, 2014	3.05 mg/dL	NA	NA	7	Post Tx - Allopurinol (300 mg/d) was started for 11 months and the current dose of allopurinol is 500 mg/d	NA	NA	NA
**8**	Quaglia et al, 2014	NA	NA	NA	7	Allopurinol started after transplantation	NA	NA	NA
		NA	1.3 mg/dL	2.3 mg/dL		Allopurinol started after transplantation (after second biopsy post transplantation)	PTx - Maintenance therapy - Tacrolimus + Mycophenolate mofetil + Steroids (sirolimus replaced by MMF after first biopsy)	NA	NA
**9**	Zaiden et al, 2014	NA	1.69 mg/dL	1.95 mg/dL	9	Post Tx - Allopurinol (mg/day) Initial dose-200, Maintenance dose- 400	After induction therapy, maintenance immunosuppression included prednisone, a calcineurin inhibitor, and Mycophenolate mofetil, or azathioprine in one case	NA	NA
		NA	2.8 mg/dL	2.48 mg/dL		Post Tx - Allopurinol (mg/day) Initial dose-100, Maintenance dose- 200		NA	NA
		NA	3.19 mg/dL	1.2 mg/dL		Post Tx - Allopurinol (mg/day) Initial dose-300, Maintenance dose- 300		NA	NA
		NA	7.63 mg/dL	2.09 mg/dL		Post Tx - Allopurinol (mg/day) Initial dose-100, Maintenance dose- 200, Febuxostat - 80 mg/day		NA	NA
		NA	4.85 mg/dL	1.8 mg/dL		Post Tx - Allopurinol (mg/day) Initial dose-150, Maintenance dose- 150		NA	NA
		NA	1.23 mg/dL	1.18 mg/dL		Post Tx - Allopurinol (mg/day) Initial dose-300, Maintenance dose- 300		NA	NA
		NA	6.78 mg/dL	NA		Post Tx - Allopurinol (mg/day) Initial dose-100, Maintenance dose- 200		NA	NA
**10**	Sharma et al, 2012	NA	3 to 4 mg/dL	NA	7	NA	NA	NA	NA
**11**	Bertram et al, 2010	NA	NA	NA	6	After one week of third transplantation dose of allopurinol doubled to 300 mg/day	PTx - IT - Basiliximab, MT - Tacrolimus (2ng/ml) + MMF (2g/day) + Steroids	Yes. Post transplantation	HLA mismatch was 1-2-1, actual panel reactive antibodies were 6%
**12**	Micheli et al, 2010	4.5	after transplantation 1.8 mg/dL, 2 weeks after Tx-3.0	2.0 mg/dL	7	Post Tx - Allopurinol started	(Post-transplant) MT-Tacrolimus + Mycophenolate mofetil + Steroid	Yes	The enzyme deficiency is due to a newly identified gene mutation in this case

	Author	Kidney function or Serum Creatinine (mg/dl)			Quality assessment score	Treatment with XOR inhibitors	Immunosuppressant treatment	Hydration and purine-based diet	Comments
		Pre (Tx) Transplant	Post (Tx) Transplant	End of follow up					
**13**	Nasr et al, 2010	11.5	1.6 mg/dL (after second transplantation)	5.5 mg/dL (after 6m), 2.6 after 10 m	7	Allopurinol 200 mg/day started after transplantation (after second biopsy post transplantation)	NA	Yes. Post transplantation	Despite subsequent resumption of allopurinol, she remained dialysis dependent thereafter
		3.9	1.3-1.4 mg/dL	2.1 mg/dL (after 4 m)		Pre Tx- 200 mg per day allopurinol which increased to 400 mg per day	NA	Yes. Pre transplantation	The patient subsequently developed multisystem organ failure and expired in 4 months post-transplant.
		7	2.5 mg/dL (1 month), 4.5 mg/dL (5 weeks),	1.6 mg/dL (18 month)		Allopurinol 300 mg twice a day after transplantation	NA	No	At 18 m PTx, the patient had stable mild chronic renal insufficiency with a serum creatinine level that had further improved to 1.6 mg/dl
**14**	Stratta et al, 2010		< 3.5 mg/dL	1.9 mg/dL	6	Allopurinol started after transplantation 300 mg/day until end of follow up of one year	NA	yes	In this patient, there was no measurable APRT activity and the allelic variant was consistent with type I APRT deficiency. However, because the patient is only heterozygous for this allele, so author infer that she is heterozygous for 2 different mutations, 1 of which we did not detect
**15**	Cassidy et al, 2004	NA	4.08 mg/dL (361 umol/l)<br><br>2.96 mg/dL (262 umol/l after induction of allopurinol)	NA	6	Allopurinol 100 mg daily (after 7 months of transplant) and increased to 300 mg daily next month	NA	Yes	It was found that both his mother and father, who are first cousins, were carriers of the condition. Of his four siblings, one brother is unaffected and two sisters are considered to be carriers with reduced erythrocyte APRT activity.
**16**	Eller et al, 2004	NA	NA	2.71 mg/dL	6	Post Tx - Allopurinol Initial dose- 150 mg x 2 /week, Maintenance dose- 100 mg/day	NA	No	In this patient 4 consecutive cadaveric renal transplantations were performed; 2,8-dihydroxyadenine crystal nephropathy recurred within weeks in the first and second graft when the patient was not treated with allopurinol immediately after transplantation. After a fourth transplantation, again without initial allopurinol, the disease recurred following an initial vascular rejection

	Author	Kidney function or Serum Creatinine (mg/dl)			Quality assessment score	Treatment with XOR inhibitors	Immunosuppressant treatment	Hydration and purine-based diet	Comments
		**Pre (Tx) Transplant**	**Post (Tx) Transplant**	**End of follow up**					
**17**	Benedetto et al, 2001	14.1 mg/dL	1.6 mg/dL	2	7	Post Tx After 19 month - 10 mg/kg allopurinol	(Post-transplant) MT- Corticosteroids + Mycophenolate mofetil + Cyclosporine	Yes	Subsequent renal biopsy failed to show disease progression
**18**	Brown et al, 1998	11.71 mg/dL (1035 umol/l)	NA	NA	7	No	(Post-transplant) Maintenance therapy- Prednisone + OKT3 + Cyclosporine	No	Post-nephrectomy she continued to deteriorate with bacterial septicemia and disseminated aspergillosis, and she died with multi-organ failure 132 days post-transplant.
**19**	De jong et al, 1996	NA	NA	NA	7	Yes (allopurinol discontinued after renal transplant)	Post-transplant cyclosporine	No	NA
**20**	Gagne et al, 1994	26 mg/dL	1.3 mg/dL	9.1	7	Allopurinol 100 mg daily (initiates after 9 years of transplant)	Post-transplant (Azathioprine and Prednisone)	Yes	NA
Induction therapy (IT), Maintenance therapy (MT), Mycophenolate mofetil (MMF), Post Transplantation (PTx), Months (m)									
